# Medicinal Chemistry Targeting Mitochondria: From New Vehicles and Pharmacophore Groups to Old Drugs with Mitochondrial Activity

**DOI:** 10.3390/ijms21228684

**Published:** 2020-11-18

**Authors:** Mabel Catalán, Ivonne Olmedo, Jennifer Faúndez, José A. Jara

**Affiliations:** 1 Clinical and Molecular Pharmacology Program, Institute of Biomedical Sciences (ICBM), Faculty of Medicine, Universidad de Chile, Independencia 1027, Santiago 8380453, Chile; mabelcatalan@u.uchile.cl; 2 Physiopathology Program, Institute of Biomedical Sciences (ICBM), Faculty of Medicine, Universidad de Chile, Independencia 1027, Santiago 8380453, Chile; ivonneolmedo@uchile.cl; 3 Institute for Research in Dental Sciences (ICOD), Faculty of Dentistry, Universidad de Chile, Olivos 943, Santiago 8380544, Chile; jvfaundez@uc.cl

**Keywords:** targeting mitochondria, delocalized lipophilic cations, mitocans, drugs, pharmacophores groups, cancer stem cells, cancer bioenergy, cancer metabolism

## Abstract

Interest in tumor cell mitochondria as a pharmacological target has been rekindled in recent years. This attention is due in part to new publications documenting heterogenous characteristics of solid tumors, including anoxic and hypoxic zones that foster cellular populations with differentiating metabolic characteristics. These populations include tumor-initiating or cancer stem cells, which have a strong capacity to adapt to reduced oxygen availability, switching rapidly between glycolysis and oxidative phosphorylation as sources of energy and metabolites. Additionally, this cell subpopulation shows high chemo- and radioresistance and a high capacity for tumor repopulation. Interestingly, it has been shown that inhibiting mitochondrial function in tumor cells affects glycolysis pathways, cell bioenergy, and cell viability. Therefore, mitochondrial inhibition may be a viable strategy for eradicating cancer stem cells. In this context, medicinal chemistry research over the last decade has synthesized and characterized “vehicles” capable of transporting novel or existing pharmacophores to mitochondrial tumor cells, based on mechanisms that exploit the physicochemical properties of the vehicles and the inherent properties of the mitochondria. The pharmacophores, some of which have been isolated from plants and others, which were synthesized in the lab, are diverse in chemical nature. Some of these molecules are active, while others are prodrugs that have been evaluated alone or linked to mitochondria-targeted agents. Finally, researchers have recently described drugs with well-proven safety and efficacy that may exert a mitochondria-specific inhibitory effect in tumor cells through noncanonical mechanisms. The effectiveness of these molecules may be improved by linking them to mitochondrial carrier molecules. These promising pharmacological agents should be evaluated alone and in combination with classic chemotherapeutic drugs in clinical studies.

## 1. Introduction

Mitochondria, often called the “powerhouses of the cell”, are essential organelles that play a well-recognized role in cell bioenergy, producing ATP via oxidative phosphorylation (OXPHOS). Mitochondria and the biochemical reactions that take place within them occupy a central position in many metabolic processes, such as the Krebs cycle, the urea cycle, and fatty acid oxidation [1]. These organelles are also involved in redox balance, calcium homeostasis, and apoptosis activation [2]. Consistent with this central role in cell metabolism, accumulating evidence suggests that dysfunctional mitochondrial bioenergetics, biosynthesis, and signaling are linked to tumorigenesis and drug resistance. Accordingly, emerging studies have begun to demonstrate that mitochondrial metabolism may be a valuable target for cancer therapy [3,4]. Solid tumors display heterogeneous characteristics, including chaotic vascularization induced by the anoxic and hypoxic zones inside the tumor. The resulting microenvironments contribute to the development of cellular subpopulations with diverse metabolic characteristics [5]. The most clinically relevant cell populations in hypoxic zones include tumor-initiating cells (TICs) or cancer stem cells (CSCs), which are highly adaptable to low oxygen concentrations [6]. CSCs are strongly dependent on ATP production via OXPHOS to survive under diverse toxic conditions, including radiotherapy and chemotherapy [7].

## 2. Mitochondria as Pharmacological Targets

Recent findings have shown that tumor cell survival in nutritionally compromised microenvironments depends critically on efficient energy production (i.e., active OXPHOS) [8]. Warburg hypothesized that mitochondrial bioenergy is defective in tumor cells [9] and that this phenomenon drives some tumor cells (those with access to nutrients) to develop a glycolytic phenotype. There is clear evidence that mitochondrial respiration is essential for tumorigenesis [10,11]. It was recently reported that tumor cells with mitochondrial DNA (mtDNA) deletions show diminished mitochondrial respiration and weak tumorigenic capacity in vivo. However, acquisition of mtDNA from normal cells was sufficient to restore tumorigenic capacity [12]. Moreover, substantial data point to the mitochondria as a major source of ATP synthesis in tumor cells. The electron transport chain OXPHOS is pivotal to energy production in tumor cells subjected to hypoxia [13].

The structure and function of the mitochondria in tumor cells differ from those of normal cells in several ways [13]. Tumor cell mitochondria show elevated glutamine metabolism and fatty acid oxidation along with decreased pyruvate oxidation, and therefore increased lactic acid production. These characteristics facilitate invasiveness, metastasis, and drug resistance [14]. Overexpression of several proteins related to energy metabolism also differentiates the mitochondria of tumor versus normal cells. Hexoquinase-2 (HK-II) is overexpressed in many types of tumors, including oral squamous cancer cells (OSCCs), and is strongly upregulated by transcription factor HIF-1α [15]. HK-II is normally located in the cell cytoplasm; however, in tumor cells, this protein adheres to the outer mitochondrial membrane (OMM), forming a complex with the voltage-dependent anion channel (VDAC) [16]. The VDAC is a mitochondrial protein located in the OMM that functions as a gatekeeper for mitochondrial molecule exchanges between these organelles and the rest of the cell, regulating mitochondrial metabolic and energy functions. The VDAC also plays a key role in mitochondria-mediated apoptosis [17]. Since ATP obtained from OXPHOS travels through the VDAC toward the cytoplasm, formation of the HKII–VDAC complex allows HK-II to use the newly-synthesized ATP to phosphorylate glucose, forming glucose-6-phosphate. Thus, HK-II activity represents the nexus between the glycolytic pathways and OXPHOS, making it an interesting pharmacological target from a bioenergetics perspective [18].

Mitochondrial structure in tumor cells is also distinct from that of normal cells. Cholesterol and cardiolipin content are present in the inner mitochondrial membrane (IMM) of tumor cell mitochondria, reducing proton permeability and significantly elevating mitochondrial transmembrane potential (∆Ψm) [19]. This difference in ∆Ψm has been exploited to develop selective mitochondrial inhibitors known as mitocans (“mitocan” is an acronym of mitochondria and cancer), including rhodamine 123 [20] and other lipophilic cations [21,22]. Research on lipophilic cations and their related synthetic molecules has shown that many mitocans exert a potent cytotoxic effect both in vivo and in vitro. This cytotoxic effect may be improved in vivo by combining these molecules with other proven chemotherapeutic agents [23,24]. Our experiments have indicated that mitocans can successfully target mitochondria. However, other authors have reported that these molecules have limited activity when used as a monotherapy. The most current data suggest that mitocans may be best used in combination with other chemotherapies, such as compounds that act on proliferating cells [25,26].

## 3. The Stem Cell Problem in Solid Tumors

It is well known that solid tumor cells have vastly heterogenous genetic and metabolic characteristics. This heterogeneity is due not only to clonal expansion of cells with different genetic compositions but also to microenvironmental variance. The microenvironment of tumor cells adjacent to blood vessels is rich in nutrients and oxygen [27]. As the distance between tumor cells and blood vessels widens, cells are subjected to hypoxia and nutrient deprivation, drastically altering their genetic and metabolic characteristics [28].

Studies on cell lineage in solid tumors have confirmed the existence of cellular subpopulations called CSCs, as noted above. These CSCs, located inside the tumor mass, have an intrinsic capacity for self-renewal, aberrant differentiation, and tumor regrowth, thus contributing to tumor heterogeneity. These CSCs reside in a microenvironment with scant oxygen levels of 1–5%. This hypoxic phenomenon underlies the growth of this population, through a mechanism related to expression of the hypoxia-inducible factor-1α (HIF-1α) [29]. As CSCs are highly resistant to chemotherapy and have the ability to easily repopulate the tumor between chemotherapy cycles, these cells represent a major problem in clinical oncology. In consequence, CSCs and/or CSC mitochondria may represent important targets in cancer treatment. Therefore, before we analyze the molecules that disrupt mitochondrial function in cancer cells, it is important to briefly review the influence of hypoxic environments on energy metabolism in tumor cells.

## 4. Energy Metabolism in Hypoxic Tumor Cells

Avascular zones of solid tumors are also characterized by nutrient deprivation. Mean glucose concentration in colon and stomach carcinoma tissues, for instance, has been measured at only a fraction of plasma concentrations [30]. Tumor cells adapt rapidly to this environment by deploying metabolic changes, such as glucose transporter overexpression (GLUT 1). This adaptive response in particular has been observed frequently in advanced stages of tumor progression [31]. Conversely, in cancer cells located near blood vessels, aerobic glycolysis plays a pivotal role in creating energy and synthesizing the metabolites necessary for rapid tumor cell proliferation (called the Warburg effect) [32]. Glycolysis, however, may be insufficient to satisfy the metabolic requirements of cancer cells under hypoxia due to the intrinsic inefficiency of glycolytic energy production, making it inadequate as an energy source in avascular tumor zones given the limited availability of glucose and oxygen. Recently, RNAi intervention techniques were used to show that mitochondrial OXPHOS, rather than glycolysis, is crucial for cell proliferation under conditions of nutrient deprivation [33]. Moreover, mitochondrial OXPHOS remains functional at oxygen concentrations as low as 0.5%, allowing the mitochondria to produce ATP under hypoxia [34].

Given the apparently crucial role of tumor mitochondria, medicinal chemistry research over the last decade has endeavored to isolate, design, synthesize, and characterize a broad number of molecules capable of selectively transporting novel or known pharmacophore groups that disturb mitochondrial function in tumor cells [35]. Many of these pharmacophore groups have been isolated from plants or chemically synthesized in the laboratory. Several drugs currently used to treat other diseases also show a capacity for targeting mitochondria. In short, there are numerous molecules of diverse chemical nature that are available for evaluation in clinical assays or in combination with classic chemotherapy in vivo.

Among the diverse molecules postulated as antitumor drugs, this review will analyze the mitochondrial and cellular effects of polyphenols, delocalized lipophilic cations, and old drugs that have demonstrated interesting results in terms of inducing tumor cell death (some in CSCs) both in vitro and in vivo. 

## 5. Polyphenols as Mitochondrial-Disrupting Agents Targeting Tumor and Cancer Stem Cells

**Figure 1:**
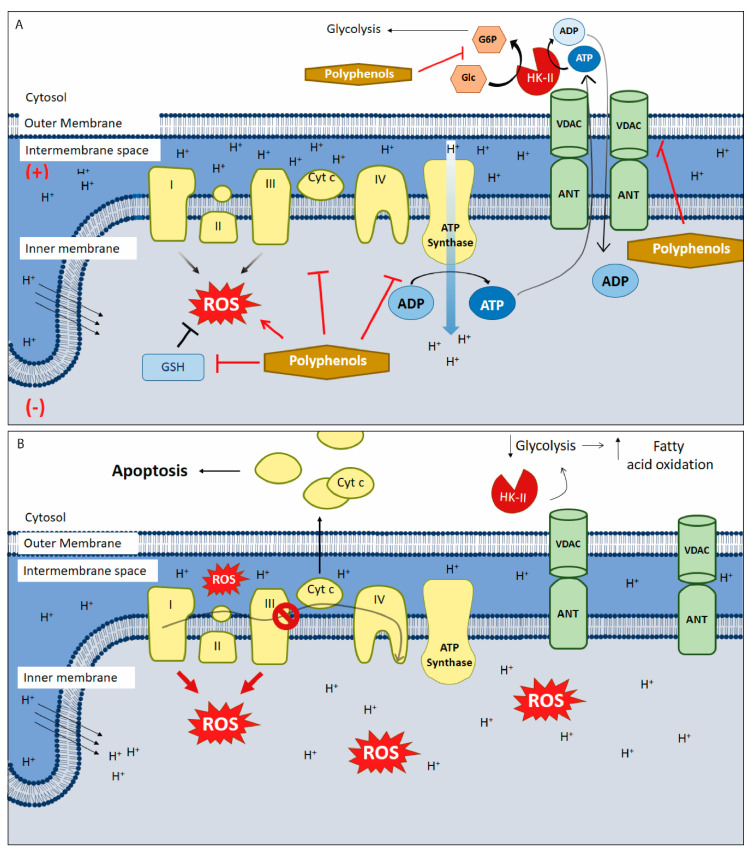
Polyphenols targeting mitochondria in cancer. (**A**) Polyphenols alters oxidative stress through the generation of intracellular reactive oxygen species (ROS) by different pathways: autoxidation, inhibiting antioxidant pathways and downregulating the modulate electron transport chain (ETC) function both directly or indirectly (e.g., by inhibiting aerobic glycolysis). They also inhibit ATP synthase, HK-II activity, and blocks the voltage-dependent anion channel (VDAC). (**B**) HK-II/VDAC complex uncoupling causing a drop in glycolysis and in the amount of ADP in the mitochondrial matrix. To compensate the lack of ATP availability, a metabolic switching occurs and metabolism changes towards fatty acid oxidation, inducing (more) ROS generation, membrane permeabilization, inner membrane depolarization and apoptosis. Furthermore, polyphenols inhibit the anti-apoptotic proteins of the bcl-2 family, allowing the exit of Cyt-c from the mitochondria triggering intrinsic apoptosis. This scheme does not include the anti-oxidant mechanisms of polyphenols. (+) positive charge in mitochondrial intermembrane space; (−) negative charge on the matrix side of the inner mitochondrial membrane; red arrows: ROS synthesis increases; inhibitory red lines: inhibitory effects of polyphenols; ↑ increases; ↓ decreases; Ø complex III inhibition.

Polyphenols are plant-derived compounds with complex chemical structures. These compounds can be classified into two main groups: flavonoids (e.g., anthocyanins, flavanols, flavanones, flavonols, flavonones, and isoflavones) and non-flavonoids (e.g., phenolic acids, xanthones, stilbenes, lignans, and tannins) [36]. Polyphenols have been studied extensively and are associated with prevention of numerous illnesses such as cardiovascular, metabolic, and neurodegenerative diseases, as well as various infections and age-related changes. All of these pathologies are related to increased oxidative stress [37]. Polyphenols have a strong antioxidant capacity, attributable to their ability to interact with free radicals generated endogenously, by radiation, or through xenobiotic metabolism, converting these molecules into less-reactive forms. Furthermore, at high concentrations (generally at the micromolar range), polyphenols may exert cytotoxic effects in diverse tumor cells [38]. Furthermore, recent research has focused on the various mechanisms of action of polyphenols as well as their capacity to target the mitochondria. These molecules appear to regulate the oxidative state of the organelles, alter ∆Ψm, and modulate electron transport chain (ETC) activity and ATP synthesis, leading to metabolic changes and, ultimately, activation of intrinsic apoptosis (Figure 1A) [39]. These responses have attracted the attention of cancer researchers due to their potential effects on tumor mitochondria. Tumor cells develop altered (and more sensitive) mitochondria to fuel tumor growth under hypoxic conditions. Experiments performed in vitro and in vivo (including in humans) have shown that polyphenols can reduce tumor size, metastasis, and cell proliferation. Additionally, these antioxidant molecules can act as chemopreventives by interrupting or preventing the carcinogenesis process [40,41].

Tumor cells have high reactive oxygen species (ROS) levels. To compensate, these cells increase expression of proteins involved in antioxidant pathways. Overexpression of these antioxidant enzymes, along with elevated ROS levels, likely underlies many of the typical features of cancer cells, including high rates of cell proliferation, metastasis, and angiogenesis, as well as resistance to chemotherapy [42]. Disrupting the increase in ROS levels during carcinogenesis can prevent cell proliferation and tumor development. During later stages of tumor promotion and progression, low ROS levels confer cancer cells with enhanced survival capacity and induce elevated ROS levels through HIF-1α stabilization, providing greater resistance to chemotherapy [43]. Inducing ROS or reducing antioxidant capacity in tumor cells under high oxidative stress has been shown to be effective against tumor progression. Thus, polyphenols are a promising target in cancer research as these molecules may act as both anti- and pro-oxidants (Figure 1B).

The catechin family of polyphenols is among the most well-known of these molecules. Catechins obtained from cacao exert pro-oxidant effects and induce apoptosis in epithelial ovarian carcinoma cell lines [44]. Other polyphenols show similar effects, increasing ROS levels in various cancer models. The body of research documenting these effects has been compiled and reviewed by Mileo and Miccadei (Table 1) [39].

As antioxidant molecules, polyphenols act as ROS scavengers, transition metal chelators, and regulators of the activity and/or expression of enzymes that participate in signaling pathways related to oxidative stress. Luteolin, a flavonoid present in apple peels, carrots, and broccoli, has been evaluated in vivo and in vitro using various breast and pancreatic cancer cell lines. In these models, luteolin induced cell apoptosis and inhibited metastasis, as well as reducing proliferation and tumor formation [45,46,47]. As an antioxidant, luteolin acted as a ROS scavenger in colon cancer cells (HT-29) and induced superoxide dismutase (SOD) and catalase (CAT) antioxidant enzyme expression. Luteolin also increased glutathione (GSH) levels and glutathione synthetase (GST) protein content. These results were accompanied by mitochondrial dysfunction, including activation of cellular apoptosis through the mitochondria-mediated caspase pathway, ∆Ψm dissipation, and decreased expression of bcl-2, an antiapoptotic protein, promoting the release of cytochrome c (Cyt c) into the cytosol. These effects were observed to be selective for colon cancer cells (HT29) and did not affect normal cells [48]. In another in vivo study using BALB/c mice in an azoxymethane (AOM)-induced colorectal cancer model (CRC), luteolin at 1.2 mg/kg body weight/day inhibited induction of carcinogenesis by increasing GST expression [49]. The same group of researchers found that luteolin also induced Nrf2 transcriptional factor expression while restoring the GSH, protein thiol (PSH), and total thiol levels that had been decreased by the AOM treatment. Moreover, luteolin inhibits translocation of β-catenin from the cytosol to the nucleus in CRC models, both in vitro and in vivo [50]. These results strongly suggest an antioxidant action of luteolin in cancer development. On the other hand, 40 µM luteolin induced ROS production, membrane permeabilization, a drop in ∆Ψm, and downregulation of the bcl-2 family of proteins in in vitro models of glioblastoma, triggering the release of Cyt c into the cytosol and activating the apoptotic pathway, as also observed in colon cancer cells (HT29) [51]. Moreover, in immune-deficient nude mice bearing U87MG tumor xenografts, 10 mg/kg luteolin, administered intraperitoneally three times per week for 35 days, inhibited U87MG tumor xenografts growth, which the authors attributed to ROS accumulation, endoplasmic reticulum (ER) stress, and mitochondrial dysfunction. Moreover, luteolin did not affect body weight, alanine aminotransferase (ALT), or aspartate transaminase (AST) activity [51].

**Table 1:** Mitochondrial targets and effects of polyphenols in cancer models.

Polyphenol	Chemical Structure	Model	Target	Consequences	Ref.
Luteolin (flavonoid)	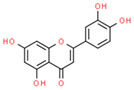	SW1990 xenograft Model	↓ bcl-2	Mitochondrial Permeabilization Cellular death ↓Tumor Growth	[47]
		HT-29	↑ GSH ↑ caspase 3 and 9	↓ ∆ψm ↓Proliferation Apoptosis	[48]
		SKM-1 Rat cancerous hepatocytes	↑ ROS ↑ caspase 3 and 9	↓ ∆ψm Apoptosis	[52,53]
Heperidin (flavonoid)	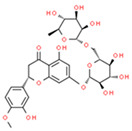	Mouse lung cancer	↑ GSH, SOD	↓Tumor Incidence, ↓PCNA	[54]
		SGC-7901, MGC-803 and HGC-27 Gastric xenograft model	↑ ROS; ↓bcl-2 ↑ Caspase 3 and 9	↓Proliferation ↓ ∆ψm Apoptosis ↓Tumor Growth	[55]
Curcumin (flavonoid)	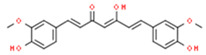	p53-deficient H1299	↓bcl-2; ↑ Bax	↓Proliferation Necrosis	[56]
		A549, SPC-A1	↑ ROS	↓ ∆ψm ↓Proliferation Apoptosis	[57]
		HCT116, HT29	↓ HK-II	HK-II mitochondrial dissociation Apoptosis	[58]
Ellagic acid (Hydroxybenzoates)	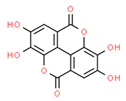	TSGH8301 SH-SY5Y	↑ Caspase 3 and 9	↓ ∆ψm ↓Proliferation Apoptosis	[59,60]
		B-lymphocytes from CLL patients	↑ ROS	Apoptosis	[53,61]
		HOP62 and H1975 Mouse lung cancer	↓ OXPHOS	↓ATP ↓Tumor Growth	[60]
Resveratrol (Silbene)	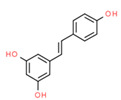	H446 TRAMP cells	↓bcl-2	↓ ∆ψm ↓ Cell Viability	[62,63]
		HeLa and MDA-MB-231	↑ ROS; ↓ GSH ↓ OXPHOS	↓Proliferation ↑ Mitophagy	[64]
Epigallocatechin Gallate (EGCG) (Catechin)	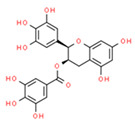	Hep2	↓bcl-2; ↑ Bax	↓ ∆ψm	[65]
		MIA PaCa-2 and SMMC7721	↑ ROS	Apoptosis	[66,67]
		SCC-25	↑ ROS	Cytotoxicity	[68]
		REN	↓ OXPHOS (I, III Complex)	↓Proliferation Apoptosis	[69]

HT29: human colon cancer cell line; SKM1: human acute myeloid leukemia cell line; SGC-7901 and MGC-803: human gastric cancer cell lines; HGC-27: human cell line derived from the metastatic lymph node of gastric cancer; p53-defficient H1299: human non-small cell lung carcinoma cell line derived from the lymph node with lack expression of p53 protein; A549: human lung(carcinoma) cell line; SPC-A1: human lung cancer cell line; HCT116: human colon carcinoma cell line; TSGH8301: human bladder cancer cell line; SH-SY5Y: neuroblastoma cell line; CLL: chronic lymphocytic leukemia; HOP62: human lung adenocarcinoma cell line; H1975: human non-small cell lung carcinoma cell line; H446: human small cell lung cancer cell line; TRAMP cells: transgenic adenocarcinoma mouse prostate cell line; MDA-MB-231: human breast cancer cell line; Hep2: human laryngeal cancer cell line; MIA PaCa-2: human pancreatic cancer cell line; SMMC-7721: human hepatocellular cancer cell line; SCC-25: human squamous cell carcinoma cell line; GSH: glutathione; ROS: reactive oxygen species; SOD: superoxide dismutase; HK-II: hexokinase II; OXPHOS: oxidative phosphorylation; (∆Ψm): mitochondrial transmembrane potential; PCNA: proliferating cell nuclear antigen. ↑ represent protein synthesis and/or activity increase; ↓ represents a decrease in the mentioned process.

In mitochondria, polyphenols can undergo transition-metal catalyzed autoxidation to produce superoxide anions (O_2_^•−^), which generate H_2_O_2_ and hydroxyl radicals. For example, EGCG (epigallocatechin gallate), a green tea catechin, can markedly increase ROS production and positively regulate expression of CTR1 (cationic transporter receptor type 1), a relevant transporter for cisplatin intake [70]. EGCG autoxidizes due to its chemical instability under experimental conditions both in vivo and in vitro, resulting in H_2_O_2_ production [71]. Polyphenols may also act as pro-oxidants by regulating the thioredoxin (Trx) pathway, which is essential for maintaining cellular homeostasis (thiol redox control) (Table 1) [72].

In parallel, polyphenols can directly regulate ETC and ATP synthase activity. French maritime pine bark extract (PBE) is a complex mixture of polyphenols, obtained from the *Pinus maritima* tree, that inhibits the ETC. This property has been demonstrated in rat liver mitochondria and submitochondrial particles, where PBE inhibits NADH-ubiquinone reductases, succinate-ubiquinone, ubiquinol-cytochrome c and, to a lesser extent, cytochrome c oxidase activity [73]. In a similar vein, Valenti et al. showed that EGCG alters mitochondrial respiratory chain complexes (I, II, and ATP synthase) in malignant pleural mesothelioma cells, causing an arrest in growth cellular, reduced ATP production via OXPHOS, mitochondrial swelling, release of Cyt c, and further induction of mitochondrial-mediated apoptosis (Figure 1A) [69].

Another recently discovered polyphenol target is HK-II, which is favorably overexpressed in the OMM of cancer cells under hypoxic conditions. HK-II complexes with the VDAC and the adenine nucleotide translocator (ANT). Polyphenols can negatively regulate or uncouple the ANT/VDAC complex, affecting HK-II function. In both cases, this uncoupling leads to downregulation of glycolysis and a metabolic shift towards beta-oxidation. Consequently, ∆Ψm is lost and apoptosis activated, inhibiting cell proliferation and tumor growth [58,74,75,76,77]. Resveratrol, a polyphenol present in black grape skins and wine, impairs the growth of non-small cell lung cancer by inhibiting HK-II activity and suppressed tumor growth in vivo in a xenograft mouse model [78]. Curcumin, the principal curcuminoid in turmeric (*Curcuma longa*), induced cytotoxicity at 20 µM in the human colorectal cancer lines HCT116 and HT29 through HK-II protein inhibition and downregulation. In addition, curcumin dissociated the HK-II complex from the mitochondria, resulting in apoptosis activation mediated by the mitochondrial pathway [58]. It was later found that 50 µM curcumin was also capable of blocking the VDAC by interacting with the N-terminal residue of this channel [44]. Other polyphenols, such as EGCG and genistein, also inhibit HK-II function, leading to apoptosis activation in human tongue carcinoma and hepatocellular carcinoma cells, respectively [75,76,77].

Overexpression of anti-apoptotic proteins, including those from the bcl-2 family, allows cancer cells to adjust to a hypoxic environment, conferring them with metabolic adaptations and resistance to intrinsic apoptosis. This phenomenon also underlies resistance to chemotherapies [79]. The polyphenols, luteolin, galangin, fisetin, and apigenin, have been shown to bind to bcl-2, inhibiting the protein [80]. In a pancreatic cancer cell model, leutonin inhibited bcl-2, inducing mitochondrial permeabilization and further apoptosis activation [47]. In addition, luteolin increases the antitumor effect of cisplatin in resistant ovarian cancer cells, inducing apoptosis activation and inhibiting cell migration and invasion [81]. AT-101, a gossypol enantiomer that mimics BH3 domains, has been used successfully as a bcl-2 inhibitor in various clinical trials, both alone and in combination with other antineoplastic agents [72]. CSCs also seem to develop resistance to apoptosis thanks to the upregulation of antiapoptotic proteins from the bcl-2 family. However, only AT-101 has been tested; this molecule showed specific apoptotic effects in CSCs [82]. Although other polyphenols have been evaluated in CSCs with favorable results [83], there are no studies showing that polyphenols specifically activate mitochondrial pathways to induce apoptosis in CSCs. Due to the importance of anti-apoptotic proteins from the bcl-2 family for CSC survival and chemoresistance, it can be speculated that other polyphenols that inhibit bcl-2 proteins (such as theaflavin and catechins, along with those mentioned above) may exert similar and specific pro-apoptotic effects on CSCs. In general, all of the previously described effects of polyphenols—reduction in ∆Ψm, induction of a metabolic switch towards fatty acid oxidation, HK-II protein inhibition, ROS generation, and ETC inhibition—may obstruct CSC invasiveness and survival (Figure 1B) [84].

Finally, polyphenols represent an attractive pharmacological alternative in cancer chemoprevention and treatment, either alone or as adjuvants, as they can induce tumor cell death and reduce the resistance of cancer cells. However, the precise mechanisms associated with mitochondrial disruption in tumor cells seem to be diverse and, in some cases, remain to be further elucidated.

## 6. New Trojan Horses for Targeting Tumor Cell Mitochondria

In addition to advances in knowledge regarding the metabolic and structural changes in mitochondria that occur during carcinogenesis, nutrient deprivation and/or hypoxia have become important foci in the search for new therapeutic targets in recent decades. Several vehicles have been developed to deliver pharmacophore groups (of natural or synthetic origin) into tumor cell mitochondria in order to induce mitochondrial damage and/or malfunction and eventually cell death. Knowledge of the particular characteristics of tumor mitochondria is critical in designing these molecules, including, as mentioned above, reduced proton permeability of the IMM and elevated ∆Ψm (induced by cardiolipin composition changes) as compared to non-tumor cell mitochondria. These characteristics suggest promising options for new pharmacological strategies.

Delocalized lipophilic cations (DLCs) are a family of cationic and lipophilic compounds that can accumulate inside the mitochondrial matrix despite the hydrophobic nature of the plasma and mitochondrial membranes, driven by negative charges in the inner face of the IMM. Some DLCs are toxic to normal cells at high concentrations, but this effect may be avoided by linking mitochondrially active pharmacophore groups to the compounds. Using this strategy, the pharmacophore groups can selectively target the mitochondria without provoking significant cell death [23,85]. The triphenylphosphonium (TPP^+^) moiety has been one of the most commonly used DLC in recent decades. Initially, this DLC was used as a molecular probe to study the relationship between ∆Ψm and OXPHOS and to measure ∆Ψm [86]. This DLC can selectivity accumulate in cancer cell mitochondria at levels >100 fold over non-tumor cells, principally because of the natural difference in ∆Ψm between cancer (ΔΨm~180–220 mV) [26,87] and normal cells (ΔΨ~140 mV) [88]. This characteristic offers an attractive target for mitochondrial-disrupting anticancer agents. TPP^+^ has also been linked to many pharmacophore groups including gallic acid, benzoic acid derivatives, various polyphenols, electrophiles, and hydrogen sulfide donors, largely with the goal of producing new antitumor or antioxidant molecules. Table 1 provides a brief overview of the diverse pharmacophore groups that have been linked to the TPP+ moiety. These compounds principally affect OXPHOS, ∆Ψm, and the mitochondrial redox state through ROS generation. Hundreds of molecules have been assessed as pharmacophore groups; for a more exhaustive analysis, see the review by Zielonka et al. [89].

**Figure 2:**
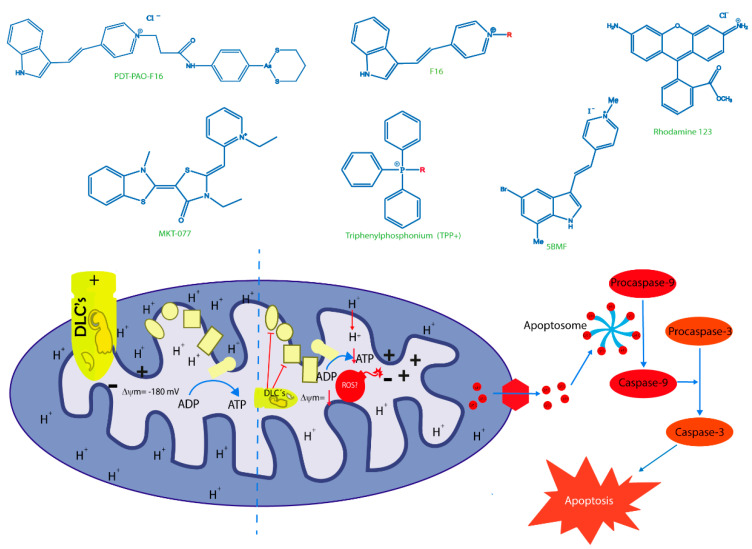
Principal delocalized lipophilic cations (DLCs) and their mechanism of action. The figure shows the chemical structures of classic lipophilic cations as Rhodamine 123, TPP+ and MKT-077. Additionally, the chemical structures of novel DLCs, such as 5BMF, F16 and their derivative PDT-PAO-F16, are shown. Lower section shows ΔΨm as the attraction force that allows all DLCs to reach tumor cell mitochondria and their respective targets described for each of them. The final effect of mitochondrial malfunctions induced by DLCs is cell death, principally through apoptosis. + positive charge in mitochondrial intermembrane space; (−) negative charge on the matrix side of the inner mitochondrial membrane; red arrows: H^+^ transposed from intermembrane space to mitochondrial matrix and a decrease in mitochondrial transmembrane potential; inhibitory red lines: inhibitory effects of delocalized lipophilic cations.

One of the first DLCs evaluated in clinical trials was MKT-077 (MKT077 or FJ-776), a rhodacyanine dye analogue that preferentially accumulates in tumor cell mitochondria. MKT-077 showed anticarcinoma activity in preclinical studies, exerting a cytotoxic effect in colon carcinoma CX-1, breast carcinoma MCF-7, pancreatic carcinoma CRL142O, bladder transitional cell carcinoma U, and melanoma LOX, but not the normal epithelial cell line monkey kidney CV-1. In vivo assays with MKT-077 showed growth inhibition of human renal carcinoma cells and human prostatic carcinoma [90]. Unfortunately, phase I clinical trials also indicated renal toxicity. Moreover, the researchers concluded that MKT-077 showed a low therapeutic index, suggesting that the drug would not be feasible as a mitochondria-targeting agent in cancer treatment [91]. Rhodamine-123 is a DLC found to be retained by prostate tumor tissue, with a maximum tolerated dose of 96 mg/m^2^. This compound was safely administered at monthly intervals without detectable drug accumulation in serum in a phase I clinical trial [92]. However, these promising results have not been reaffirmed in new phase II and III clinical trials. F16 ((*E*)-4-(1 *H*-indol-3-ylvinyl)-*N*-methylpyridinium iodide)) is a fluorescent DLC that was first screened as a new antiproliferative agent. The cytotoxic activity displayed by F16 could be attributable to mitochondrial dysfunction, associated with induction of mitochondrial permeability transition pore (mPTP) opening [93]. However, new publications showed that both F16 and PVI ((*E*)-3-(2-(pyridine-4yl) vinyl)-1 *H*-indole), the uncharged precursor compound used to synthesize F16, induced an uncoupling effect in tumor cell mitochondria. Moreover, F16 selectively accumulated in mitochondria and induced a striking toxicity in tumor cells. When both compounds were directly incubated with isolated mitochondria, the toxic effects of F16 and PVI were quite similar. In particular, both compounds caused ultrastructural changes, swelling, increased inner membrane permeabilization, and altered membrane fluidity in the mitochondria. Moreover, PVI also acted as an uncoupler, affecting mitochondrial respiration in a manner similar to that of F16, provoking decreased intracellular ATP levels, ΔΨm dissipation, and Cyt c release, indicating mPTP opening [94]. Nevertheless, the therapeutic efficacy of F16 has not been sufficient for the compound to be considered a prevailing candidate for drug development. Structural modifications of the F16 compound have resulted in limited improvements to its anticancer effects [95], with in vivo results similar to those described for old cytotoxic drugs (Figure 2) [96].

Some authors posit that DLCs have inherently limited anticancer activity as they often fail to cause a large-scale disruption of ΔΨm, falling short of decreasing ATP production and killing the cell. To date, DLCs have been used principally as a cargo group to deliver selectively functional molecules to the mitochondria (Table 2). Researchers have synthesized F16 derivatives with substitutions in the indole ring of F16 to produce molecules with improved selectivity and antitumor activity, such as a 5BMF derivative that displays cytotoxic effects in vitro and antitumor effects in vivo. Furthermore, 5BMF has been useful as a fluorescent probe for tumor imaging capacity [97]. This molecule was complexed with human serum albumin (HSA) to form the 5BMF@HSA complex, which improved the fluorescence intensity of 5BMF and increased its solubility by nearly 3.4-fold. Moreover, the complex showed cytotoxic effects in the human glioblastoma cell line U87MG and breast cancer cell line MDA-MB-231 at the µM range, and no clear selectivity for non-tumor cells. Both 5BMF and 5BMF@HSA produced a decrease in subcutaneous U87MG tumors, but the complex showed slightly better tumor growth suppression than 5BMF group alone. Thus, the 5BMF@HSA complex has potential applications in both cellular imaging and antitumor therapeutics [97].

Liu et al. assessed the organic arsenical F16 derivative DPT-PAO-F16 as a fluorescent mitochondria-targeting anticancer molecule (Figure 2) [98]. Based on reports regarding arsenic trioxide in acute promyelocytic leukemia (APL), the authors synthetized DPT-PAO-F16 to enhance anticancer activity and visualize distribution inside the cells. They found that PDT-PAO-F16 mainly accumulated in the mitochondria and inhibited viability more effectively than F16 alone. In addition, PDT-PAO-F16 inhibited both the pyruvate dehydrogenase complex (PDHC) and respiratory chain complexes. These researchers concluded that introduction of the F16 moiety to the arsenical organic compound improves anticancer potency and selectivity, as it accumulates in cancer cell mitochondria as a lipophilic cation. PDT-PAO-F16 mainly inhibited the activity of PDHC and respiratory chain complexes I and IV, leading to mitochondrial dysfunction and depolarization, ROS generation, suppression of ATP synthesis, and disordered thermogenesis. Membrane swelling and enhanced permeability were thus triggered, resulting in Cyt c release and activation of caspase-dependent ROS-mediated apoptosis. Finally, PDT-PAO-F16 has also been shown to inhibit APL in a mouse model (Figure 2) [98].

Lately, interest in another DLC has been renewed—the cyanine dye derivative D112, discovered in the 1970s, which shows toxicity against colon cancer cell lines [99]. Recently, D112 treatment has been found to induce caspase activation, mitochondrial depolarization, and phosphatidylserine externalization in the T-cell leukemia cell line, Jurkat, leading to apoptosis induction. Moreover, studies have indicated that D112 can accumulate in tumor cell mitochondria, with increased toxicity in tumor versus non-tumor cells [100]. Additionally, the same research group found that D112 inhibits electron flow through the ETC, leading to ROS production, mtDNA damage, bax protein activation, and apoptosis induction. Furthermore, Gopping et al. showed that photo-activation is selectively improved in tumor cells and that D112 has the capacity to induce ROS generation, either by direct transfer of electrons to oxygen or via ETS-mediated ROS generation, which is less tolerated by tumor cells, ultimately triggering bax-dependent apoptotic pathways [101].

## 7. Old Drugs Targeting Tumor and Cancer Stem Cell Mitochondria

In recent decades, there has been a growing interest in identifying possible antitumoral effects of drugs previously approved for other clinical indications. Consequently, we will review the most relevant drugs with mitochondrial activity.

**Figure 3:**
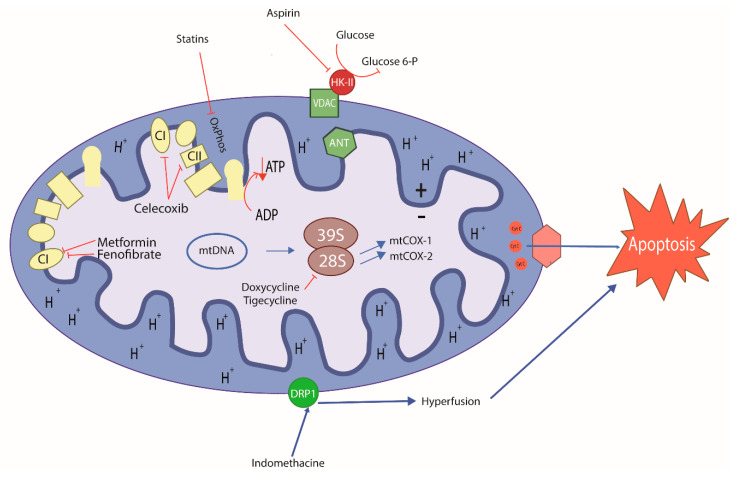
Schematic mode of action of old repurposing drugs in cancer cell. Antibiotics, anti-inflammatories, and metabolic control drugs can target mitochondria inducing metabolic stress, leading to cancer cell and CSC death. Doxycycline and tigecycline are antibiotics; acetylsalicylic acid, celecoxib and indomethacin are anti-inflammatories; fenofibrate, simvastatin and lovastatin are antilipemic; metformin is antiglycemic. (+) Positive charge in mitochondrial intermembrane space; (−) negative charge on the matrix side of the inner mitochondrial membrane; blue lines: induced effects; inhibitory red lines: inhibitory effects of repurposing drugs; CI: Mitochondrial Complex I; CII: Mitochondrial Complex II.

Several classes of existing drugs used to treat inflammation, infections, metabolic diseases, and other problems have been shown to exert antitumor effects, especially as mitochondrial target agents. Many of these agents induce severe energy stress by targeting the mitochondria, eventually triggering cell death (Table 3) [86]. Within the wide range of mitochondria-targeting agents with antitumor potential, specific drugs are especially interesting due to their actions on CSCs (Figure 3) [102,103].

Antibiotics are one such promising pharmacological group. Many of these drugs have a singular chemical structure that allows the agents to target mitochondria and induce a pharmacological effect, often inhibiting mitochondrial protein translation. These effects may also occur in mitochondria due to similarities between this organelle and bacteria [104,105]. These drugs include those that act on the 50s ribosomal subunit of bacterial ribosomes, such as macrolides, and inhibitors of the 30s subunit, such as tetracyclines. Lamb et al. described for the first time that antimicrobial agents selectively induced cytotoxic effects in several types of tumor cell lines by inhibiting mitoribosomal subunits, 39S and 28S, which are homologous to bacterial subunits. The authors emphasized the actions of these agents in CSCs, as these cells depend on mitochondrial protein biogenesis to remain viable (Table 3) [106].

**Table 3:** Pharmacophore groups linked to TPP+ targeting mitochondria.

Name	Pharmacophore	Mitochondrial Target	Year	References
CoQ10	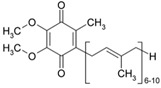	OXPHOS	2000	[107]
α-Tocopherol	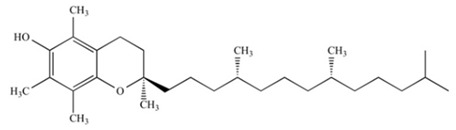	OXPHOS	2004	[108]
Gallic Acid	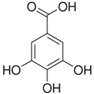	ΔΨm, uncoupling effect	20142017	[23,109]
Doxorubicin	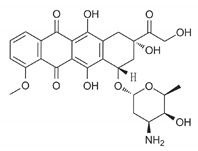	ROS generation	2014	[110]
F16	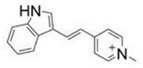	ΔΨm	2014	[95]
Chlorambucil	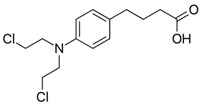	mtDNA	2013	[111]
DNP (2,4-dinitrophenol)	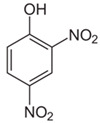	ΔΨm	2006	[112]
Lonidamide	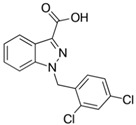	OXPHOS (complex I)	2019	[113]
Metformin	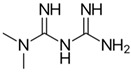	OXPHOS (complex I)	2016	[114,115]
Paraquat	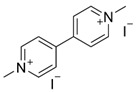	ROS generation	2020	[115]
Artemisinin	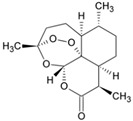	ΔΨm	2017	[116]
Curcumin	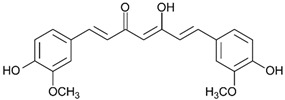	ROS generation, ΔΨm, AKT inhibition and STAT3 phosphorylation	2014	[117,118]
Benzoic acid derivatives	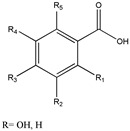	OXPHOS uncoupling effect	2016	[26]

OXPHOS: oxidative phosphorylation; ΔΨm: mitochondrial transmembrane potential; ROS: reactive oxygen species; mtDNA: mitochondrial DNA.

The antibiotic most widely known as an antitumor agent is doxycycline. This drug is mainly used to either disrupt mitochondrial protein generation through accumulation in the mitochondrial matrix of human cells or to inhibit the 28S mitoribosomal subunit. Recently, Protasoni et al. [119] reported that doxycycline exerts cytotoxic effects in lung and colon tumor cells, noting that the drug exclusively inhibited translation of mitochondrial proteins such as cytochrome c oxidase subunits 1 and 2 (mtCOX1 and mtCOX2, respectively), as described later by Dijk et al. [120]. Additionally, doxycycline in combination with azithromycin decreased intracellular ATP levels in breast cancer cells by inhibiting cellular respiration, eradicating CSCs and provoking catastrophic energy stress that impeded cell survival [121]. Many studies suggest that these mitochondrial stressors may lead to metabolic alterations in the cell that could trigger apoptosis in various cancers, such as leukemia and melanoma [120,122,123]. Furthermore, the Ferreira research group recently reported that doxycycline combined with lipophilic cations derived from gallic acid and gentisic acid enhanced cytotoxic and antineoplastic activity in vitro and in vivo, respectively, in syngeneic breast cancer models. Doxycycline was also shown to decrease mtCOX1 and mtCOX2 expression in breast cancer cells. Moreover, this drug decreased mitochondrial mass and increased peroxisome proliferator-activated receptor-γ coactivator 1α (PGC-1α) levels as a compensatory effect in vivo in a human mammary carcinoma model. However, the effects of doxycycline on CSCs have not yet been explored by these authors [109,124]. Based on these preclinical findings, Scatena et al. conducted the first clinical trial of doxycycline, administering the drug to a small group of patients with early-stage breast cancer. The authors reported that doxycycline decreased CD44^+^ positivity, a CSC marker, suggesting the possible efficacy of this drug in resistant breast cancers [125]. Zhang et al. had also previously described a decrease in CD44^+^ positivity in breast cancer cells, accompanied by reduced numbers of CSCs and subsequent inhibition of cell proliferation [126].

Tigecycline, a glycylcycline from a new group of antibiotics, has been approved for intravenous administration. Similar to tetracyclines, this drug has been described as an antineoplastic agent that acts by inhibiting the 28S mitoribosomal subunit in many types of cell lines. Tigecycline was shown to inhibit cellular respiration in thyroid cancer, reducing mtCOX1 and mtCOX2 translation, lowering ATP levels, and inducing cell death due to energy stress in a manner comparable to doxycycline [127]. Similar findings have been described in many cancer cell types such as leukemia, lymphoma, and lung cancer [128,129,130,131]. In chronic myeloid leukemia, tigecycline selectively targeted CSCs, leading to inhibition of OXPHOS by disturbing mitochondrial function, potentially leading to apoptosis in cells dependent on adequate mitochondrial oxidative metabolism for survival [131].

Another group of drugs with potential as antineoplastic agents are non-steroidal anti-inflammatory drugs (NSAIDs). NSAIDs have been investigated extensively, with many studies describing mechanisms that might inhibit cell proliferation. There is some evidence that NSAIDs act on the mitochondria to induce antineoplastic effects. The classic mechanism of NSAIDs as anti-inflammatory agents occurs through inhibition of the enzyme cyclooxygenase, which blocks production of inflammatory prostaglandins and thromboxane [132]. The antineoplastic actions reported have been described primarily for acetylsalicylic acid (ASA). ASA may contribute to mitochondrial oxidative stress, inducing apoptosis in tumor cells such as human colorectal, breast, and hepatic cancers [133,134]. Mitochondrial calcium has been found to be essential for an ASA-induced mitochondrial effects in many different types of cancer cells [135]. Recently, Tewari et al. reported that ASA interacts directly with VDAC, a protein that is overexpressed in most cancers. VDAC modulation seems to trigger apoptosis, allowing for a reduction in ΔΨm and subsequent release of Cyt c. In addition, it has been found that ASA closes the VDAC, a phenomenon that interferes with the normal flow of metabolites and ions. This alteration allows for an elevated influx of calcium into the mitochondria, triggering cell death in cervical cancer cells due to calcium overload [136]. It has also been reported that ASA induces apoptosis by increasing bcl-xl and bax while decreasing bcl-2 in colon cancer [137]. However, the effect of ASA on CSC has been described as a product of its classical mechanism of action rather than a mitochondrial mechanism. Chen et al. reported that the efficacy of ASA in lung cancer cells is attributable to decreased cyclooxygenase type 2 activity under hypoxic conditions [138]. Another group reported that the potential antineoplastic mechanism of ASA in colorectal cancer cells is due to acetylation of the histone H3K9, inducing FasL expression and selectively inducing CSC death [139]. In addition, the selective cyclooxygenase 2 inhibitor, celecoxib, has been demonstrated to function as an antineoplastic agent. It was recently reported that celecoxib inhibits ETC complexes I and III, consequently reducing oxygen consumption, triggering an increase in superoxide anions and leading to apoptosis in various metastatic cell lines, such as murine melanoma and human breast cancer [140]. Other NSAIDs, such as indomethacin, have also been shown to display antineoplastic activity. Interestingly, indomethacin can inhibit proteasomal activity and trigger mitochondrial dysfunction, leading to cell death in ovarian and lung cancer [141]. In addition, this drug may be involved in mitochondrial dynamics through a mechanism that generates PKC-ζ activity, leading to dynamin-related protein 1 (DRP1) stimulation, promoting mitochondrial hyperfission, and ultimately triggering cell apoptosis in gastric cancer (Table 3) [142].

Other mitochondria-targeting agents with antineoplastic effects include drugs used to treat metabolic diseases, such as diabetes and hyperlipidemia medications. Antilipemic agents such as fibrates and statins first showed a capacity to trigger cell death by acting on the mitochondria. Fibrates can activate the nuclear peroxisome proliferator-activated receptor α (PPAR-α), reducing triglyceride-type fatty acid levels in adipocytes and muscles, to exert an antineoplastic action through a mitochondrial mechanism [143]. Fenofibrate, a type of fibrate, could induce apoptosis by accumulating within the mitochondria. In a neuroblastoma cell model, this drug inhibited ETC complex I through a PPAR-α-independent mechanism, producing a PPAR-α-mediated metabolic shift from glycolysis towards beta-oxidation of lipids. This change depleted the cells of ATP, reducing ΔΨm and provoking cell death [144]. In gastric tumor cells, paradoxically, the mechanisms that lead to bioenergetics reprogramming have been shown to depend on the direct action of fenofibrate on its PPAR-α receptor. This action triggers a decrease in glycolysis and beta-oxidation, indirectly provoking apoptosis by increasing mitochondrial ROS [145]. However, the mechanism of action on CSCs remains unknown.

Statins are another type of antilipemic drug with proven antitumor effects. These drugs inhibit the enzyme 3-hydroxy-3-methylglutaryl-coenzyme A (HMG-CoA) reductase, which is the rate-limiting step for cholesterol synthesis in the mevalonate pathway. Normally, statins act on the muscle by provoking mitochondrial oxidative stress, affecting the function of the organelle. These effects may cause myalgia or rhabdomyolysis—well-documented and characteristic side effects of these drugs [146]. It has recently been reported that short-term exposure to the statins simvastatin and lovastatin may reduce ΨΔm in hepatic and lung cancer models, leading to decreased ATP levels through OXPHOS inhibition, independent of cholesterol synthesis. A partial increase in glycolysis due to OXPHOS inhibition has also been reported for another agent with the same mitochondrial mechanism [147]. Furthermore, statins have been shown to act on CSCs by inhibiting geranylgeranyl of RhoA, which is a characteristic consequence of mevalonate pathway inhibition [148]. Other studies have shown that lipids can control the proliferation status of colorectal CSCs through the MAPK-dependent pathway, offering a molecular target for statins [149]. The effects of statins on CSCs that are attributable to mevalonate pathway disruption have been reviewed previously [150,151,152].

Metformin is a hypoglycemic drug with proven antitumor activity. This biguanide has been shown to act on various types of tumors as described in several epidemiological, preclinical, and clinical studies [153]. The common mechanism of action of metformin is activation of 5′-AMP-activated protein kinase (AMPK), which is a master metabolic sensor. In diabetes, this mechanism could trigger optimization of energy consumption, increasing glucose uptake and glycolysis [154]. Metformin is thought to exert its anticancer effects by inhibiting OXPHOS, especially through non-competitive inhibition of ETC complex I [155]. This effect subjects the tumor cells to energy stress that triggers cell death by apoptosis [156,157]. Interestingly, metformin has been shown to have selective efficacy on CSCs in breast cancer, sensitizing the cells to standard therapies [158,159]. It has also recently been reported that upregulation of OXPHOS genes in breast cancer cells enhances resistance to metformin, while cells with elevated glycolytic gene expression are more sensitive to the drug. This finding that could partly explain the efficacy of this drug in CSCs, as these cells reside in nutrient-scarce, hypoxic environments, increasing their chemoresistance [156]. The effects of metformin on CSCs were exhaustively reviewed by Shin et al. [154]. Combinations of metformin and breast cancer drugs, such as trastuzumab, have shown particular efficacy in increasing disease-free and overall survival in diabetic patients with early-stage HER2^+^ breast cancer in a phase III clinical study [160], suggesting that metformin improves antitumor therapy in breast cancer. The body of clinical studies on metformin has been reviewed extensively by Chae et al. [161]. It should be noted that metformin did not produce significant effects as a monotherapy in clinical studies. Therefore, this drug is described as an adjuvant to standard chemotherapy.

## 8. Concluding Remarks and Future Prospects

Mitochondria have emerged over the past decade as a promising target for new and old drugs known to selectively disturb the function of this organelle. Numerous tumor models, including in vivo and in vitro studies, have demonstrated the potential of this approach. Various molecules with diverse chemical properties have also been identified as potential pharmacophore groups or vehicles to deliver drugs to mitochondrial targets. Polyphenols, largely derived from plants, are of particular interest, as these molecules may induce mitochondrial dysfunction. Furthermore, several old drugs have been repositioned as mitochondriotropic molecules. Finally, some promising data have emerged regarding new lipophilic cations, such as F16. This fluorescent probe shows interesting cytotoxic activity, but F16 has not yet been sufficiently explored as a vehicle for novel pharmacophores.

We now know that metabolic changes triggered by the tumor microenvironment elicit the presence of troublesome cell subpopulations, including CSCs. These cell populations pose significant challenges for medicinal chemistry, along with all of the previously identified metabolic characteristics of cancer cells that confer them with resistance to chemotherapy. The cytotoxic effects of many mitochondria-targeting anticancer agents have been demonstrated using normoxia models, in which CSC subpopulations tend to be small. In the years ahead, it will be important to confirm whether these agents are also effective against CSCs, which typically emerge in greater numbers as oxygen becomes scarcer. It may be especially useful to develop mitocans that can block induction of resistance mechanisms, as these molecules may be capable of inducing energy stress that inhibits mitochondrial function, provoking tumor cell death through autophagy or other processes.

Another crucial task will be to research the effectiveness of mitocans in clinical trials and to explore drug combinations that improve antineoplastic activity without increasing adverse effects. New F16 fluorescent derivatives or other DLCs may enhance the cytotoxic activity of these drugs, even against CSCs. If such agents can be linked to potent mitochondria-targeting molecules found to exert antineoplastic effects in clinical trials, mitocans may become an applied therapy for patients with various types of cancers.

Finally, in addition to the search for better and safer mitocans, it will be equally important to seek new combinations of drugs already approved for clinical use that produce synergistic antineoplastic effects.

## Figures and Tables

**Table 2:** Summary of most important old repurposing drugs for cancer stem cells.

Drug	Family Drug	Chemical Structure	Mechanism of Action	Effects in CSC	Mitochondrial Mechanism
Doxycycline	Tetracycline/Antibiotics	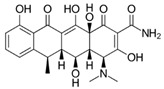	30S ribosomal subunit inhibition in bacteria	↓OXPHOS protein translation leading to OXPHOS inhibition	28S mito-ribosomal subunit inhibition
Tigecycline	Glycylglycine	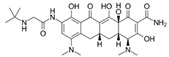	30S ribosomal subunit inhibition in bacteria	↓ OXPHOS proteins translation leading to OXPHOS inhibition	28S mito-ribosomal subunit inhibition
Acetylsalicylic acid	Salicylates/NSAIDs	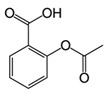	Inhibition of COX1 and COX2	Inhibition COX-2. Histone H3K9 acetylation, leading to FasL-mediated apoptosis	VDAC inhibition
Indomethacin	Indole derivative/NSAIDs	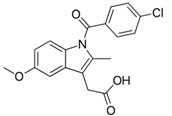	Inhibition of COX1-COX2	Apoptosis through mitochondrial hyper fission. Proteosome inhibition	Activation of DRP1
Celecoxib	Pyrazole derivative/NSAIDs	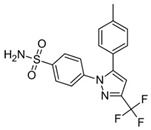	Selectively inhibition of COX2	OXPHOS inhibition	Inhibition of Complex I and III from ETC
Fenofibrate	Fibrate/Antilipemic	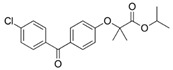	Activation of PPAR-α Receptor	OXPHOS inhibition	Inhibition of Complex I from ETC
Simvastatin	Statin/Antilipemic	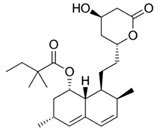	HMG CoA reductase inhibition	OXPHOS inhibition	Unknown
Lovastatin	Statin/Antilipemic	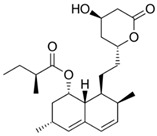	HMG CoA reductase inhibition	OXPHOS inhibition	Unknown
Metformin	Biguanide/Anti-hyperglycemic	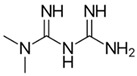	Activation of AMPK	OXPHOS inhibition leading to AMPK activation	Inhibition of Complex I of ETC

OXPHOS: oxidative phosphorylation; COX1: cyclooxygenase isoform 1; COX2: cyclooxygenase isoform 2; VDAC: voltage-dependent anion-selective channel; DRP1: dynamin-related protein 1; ETC: electron transport chain; PPAR-α: peroxisome proliferator-activated receptor α; HMG CoA reductase: 3-hydroxy-3-methyl-glutaryl-CoA reductase; AMPK: AMP-activated protein kinase. ↓ protein translation decrease.
